# Functionalized MXene (Ti_3_C_2_T_X_) Loaded with Ag Nanoparticles as a Raman Scattering Substrate for Rapid Furfural Detection in Baijiu

**DOI:** 10.3390/foods13193064

**Published:** 2024-09-26

**Authors:** Jian Chen, Xiaoyu Cao, Wei Liu, Jianghua Liu, Liang Qi, Minmin Wei, Xuan Zou

**Affiliations:** 1 School of Food Science and Engineering, Shaanxi University of Science and Technology, Xi’an 710021, China; 2 School of Biological and Pharmaceutical Sciences, Shaanxi University of Science and Technology, Xi’an 710021, China

**Keywords:** SERS, MXene (Ti_3_C_2_T_x_), Ag nanoparticles, furfural, rapid detection

## Abstract

Furfural is an essential compound that contributes to the distinctive flavor of sauce-flavored Baijiu. However, traditional detection methods are hindered by lengthy and complex sample preparation procedures, as well as the need for expensive equipment. Therefore, there is an urgent need for a new approach that allows rapid detection. In this study, we developed a novel surface-enhanced Raman spectroscopy (SERS) substrate by constructing MXene (Ti_3_C_2_T_X_) @Ag nanoparticles (Ag NPs) through an electrostatic attraction method. The MXene (Ti_3_C_2_T_X_) @Ag NPs were successfully fabricated, with adsorbed NaCl-treated Ag NPs uniformly absorbed on the surface of MXene (Ti_3_C_2_T_X_), creating high-density distributed SERS “hot spots”. The prepared substrate demonstrated excellent sensitivity, uniformity, repeatability, and long-term stability, with a low detectable concentration of 10^−9^ M for R6G (Rhodamine 6G) and an enhancement factor of up to 7.08 × 10^5^. When applied for the in situ SERS detection of furfural in Baijiu, the detection limit was as low as 0.5 mg/L. Overall, the proposed method offers rapid, low-cost, and sensitive quantitative analysis, which is significant not only for detecting furfural in Baijiu but also for identifying hazardous substances and distinguishing between authentic and counterfeit Baijiu products.

## 1. Introduction

SERS has been widely applied in materials science, chemistry, and life sciences due to its non-destructive nature, high sensitivity, and rapid detection capabilities [1,2,3]. There are two main mechanisms for the amplification of SERS signals, which are electromagnetic and chemical enhancement [4,5,6]. The amplification of SERS signals is primarily attributed to the enhancement of the local electromagnetic field induced by the surface plasmon resonance of metal nanoparticles [7]. Additionally, short-range chemical enhancement is mainly associated with the polarizability of molecules, which depends on the electronic interactions between the target molecules and the noble metal nanostructures [8]. Typically, metallic structures such as gold or silver nanoparticles are commonly applied in SERS substrates because of their high enhancement factors, ranging between 10^5^ and 10^6^ [9]. Compared to gold nanoparticles (Au NPs), Ag NPs demonstrate superior SERS performance, making them a more optimal platform for detection [10]. However, the poor stability and uniformity of metal nanostructures can limit the practical applications of noble metal substrates. As a result, there is a significant need to develop substrates that not only provide high enhancement capabilities, but also offer higher stability and uniformity. 

To tackle the challenge of enhancing SERS signals, researchers have explored a wide range of materials with promising SERS activity. Among these, two-dimensional materials, particularly MXene, have become prominent subjects of investigation. MXene is characterized by the structural formula of M*_n_*_+1_X*_n_*T_x_, where M represents an early transition metal, X represents carbon and/or nitrogen, and T_x_ represents surface chemical terminal groups [11,12,13]. Specifically, Ti_3_C_2_T_x_ is considered an ideal SERS substrate due to its abundant surface electrons, metal-like band structure, and flat surface area [14,15,16]. The enhanced Raman scattering effect of MXene (Ti_3_C_2_T_x_) is primarily chemical enhancement chemical enhancement via photoelectron-induced charge transfer, although this effect is generally weaker than electromagnetic enhancement [17,18]. To further enhance the SERS performance of MXene (Ti_3_C_2_T_x_) materials, a promising approach involves creating hybrid systems by incorporating noble metal particles. This strategy addresses the long-term stability issues of Ag NPs and enhances the relatively weak Raman signal of MXene (Ti_3_C_2_T_x_). For instance, Seong Soo Yoo et al. [19]. developed a strategy to fabricate a SERS substrate by conformally coating the surface of Ag NPs with MXene (Ti_3_C_2_T_x_), achieving an impressive analytical enhancement factor of up to 1.6 × 10^10^. This composite substrate was further functionalized to enable the highly sensitive detection of chromium ions, with a detection limit as low as 13 ng/L. Similarly, Yang et al. [20] employed a self-reduction method to synthesize Ag nanoparticles on MXene (Ti_3_C_2_T_x_), achieving an enhancement factor of 1.33 × 10^6^ and a detection concentration as low as 10^−8^ M for the probe molecule R6G. In summary, hybrid materials combining metal particles with MXene (Ti_3_C_2_T_x_) show significant potential in SERS applications due to their excellent Raman signal enhancement.

Baijiu is a traditional Chinese distilled spirit prepared from grains and cereals [21] that is historically renowned in China and characterized by twelve different flavor types [22]. According to data from the China Wine Industry Association, the Baijiu industry in China produced a total output of 6.29 million kL and generated sales revenue of 756.3 billion yuan. Among these flavor types, four dominant aromas, including soy sauce, rice, strong “cellar”, and light described as fruity and floral, are particularly dominant in Baijiu [23,24]. Furthermore, Baijiu contains numerous aromatic compounds, with furfural being one of the most significant compounds in sauce-flavored Baijiu. Furfural is formed through the decomposition of pentose at high temperatures [25]. By quantifying the furfural content in Baijiu, the relationship between furfural content and the flavor of Baijiu can be explored. The fermentation temperature and time influence the furfural content, resulting in variations across different Baijiu flavor types. For instance, sauce–flavored Baijiu generally has much higher furfural content than other flavor types [26]. Furfural contributes significantly to the aromatic profile of sauce-flavored Baijiu, with typical concentrations usually less than 450 mg/L [27]. Nonetheless, high concentrations of furfural can pose potential health risks [28]. Generally, furfural in food has been detected using high-performance liquid chromatography (HPLC), fluorescence spectroscopy, and liquid chromatography–mass spectrometry (LC-MS) [29,30,31]. However, these methods often require complex operations, expensive equipment, and an extended analysis time. Furthermore, due to the precious nature of Baijiu samples, non-destructive detection methods are preferred, a requirement that SERS can fulfill. Additionally, SERS offers other advantages, such as rapid detection, ultra-sensitivity, and simple operations [32]. For instance, Lei et al. [33] developed a SERS filter membrane modified with 4-ATP on CNTs@NiO-Fe_2_O_3_-AgS to detect furfural in mineral oil, achieving a detection limit as low as 0.025 mg/L. Similarly, Wan et al. [34] fabricated flower-like silver nanoparticles modified with carbon nanotubes (CNTs@Ag-F-AgNPs) as a SERS substrate for detecting furfural in transformer oil, achieving a detection limit of 2.25 mg/L and good reproducibility. Although there are several reports of furfural detection using SERS, most studies have focused on transformer oil, with limited application of SERS for detecting furfural in Baijiu.

Based on these factors, we proposed constructing MXene (Ti_3_C_2_T_x_) @Ag NPs using an electrostatic self-assembly method. A negatively charged MXene (Ti_3_C_2_T_x_) was modified with Poly (diallyl dimethylammonium chloride) (PDDA), a cation polymer, which converted the MXene (Ti_3_C_2_T_x_) surface charge to positive. This surface modification facilitated the uniform loading of NaCl-treated Ag NPs onto the surface of the MXene (Ti_3_C_2_T_x_). Subsequently, the aggregation agent, PDDA concentration, and the ratio between MXene (Ti_3_C_2_T_x_) and Ag NPs were optimized. Under these optimized conditions, the sensitivity of the MXene (Ti_3_C_2_T_x_) @Ag NP substrate was evaluated, with the detection limit for R6G down to 10^−9^ M. This high sensitivity was attributed to the electromagnetic enhancement from nearby Ag NPs and the charge transfer among MXene (Ti_3_C_2_T_x_), Ag NPs, and the target molecule. The substrate also demonstrated excellent repeatability, uniformity, and stability. Finally, this SERS substrate was employed for detection of trace amounts of furfural in Baijiu, showing a strong linear correlation between furfural concentration and Raman intensity. The proposed detection method is valuable for accurately distinguishing between authentic and counterfeit Baijiu, making it potentially applicable to other food products. Additionally, the detection of furfural in Baijiu is beneficial for researchers and professionals studying and analyzing Baijiu’s flavor.

## 2. Materials and Methods

### 2.1. Chemicals

Silver nitrate (AgNO_3_, ≥99.8%), sodium chloride (NaCl, ≥99.5%), and PDDA (20 wt%) were purchased from Shanghai Maclin Biochemical Technology Co., Ltd. (Shanghai, China). Potassium iodide (KI, ≥99.19%) was sourced from Shanghai Bichen Biochemical Technology Co., Ltd. (Shanghai, China). R6G (≥99.0%) was obtained from Sigma Aldrich Co., Ltd. (St. Louis, MO, USA). A few-layer dispersion of MXene (Ti_3_C_2_T_x_) was supplied by Jiangsu Xianfeng Nanomaterial Technology Co., Ltd. (Nanjing, Jiangsu, China). Furfural (99.9%) was purchased from Beijing Tanmo Quality Inspection Technology Co., Ltd. (Beijing, China). Deionized water was used in all experiments. Light-flavored Baijiu and sauce-flavored Baijiu were obtained from the local supermarket. All chemicals were used directly without further purification.

### 2.2. Instruments

All Raman spectra were collected using a laser confocal Raman microscope (DXR3 Thermo Fisher Scientific, Waltham, MA, USA). Material morphologies were investigated with a scanning electron microscope (SEM) (Apreo 2, Thermo Fisher Scientific, USA) at 20 kV with a working distance of 10.1 mm and transmission electron microscope (TEM) (Talos F200X, Thermo Fisher Scientific, USA) at 200 kV. The zeta potential of the materials was measured using a zeta potential analyzer (Litesizer 500, Anton Paar GmbH, Graze, Austria). UV–visible absorption spectra were recorded using a spectrophotometer (Evolution 201, Thermo Fisher Scientific, USA).

### 2.3. Methods

#### 2.3.1. Synthesis and Cleaning of Ag NPs 

Ag NPs were synthesized using the classical sodium citrate reduction method [35], resulting in residual citrate on the surface of the Ag NPs, potentially reducing subsequent detection sensitivity. Therefore, it is essential to thoroughly rinse Ag NPs with inorganic salts to obtain a purer substrate. In this study, Ag NPs were treated with two inorganic salts, NaCl and KI. Briefly, 9 mg of silver nitrate was added to deionized water at 45 °C and heated to boiling. Then, 2 mL of 1% sodium citrate solution was added while stirring. The solution was stirred continuously for 60 min and allowed to cool. 

The resulting colloidal Ag NPs solution was treated with inorganic salts. NaCl was dissolved in deionized water at concentrations of 1.3 M, 0.1 M, 0.09 M, 0.05 M, and 0.01 M. KI was dissolved in deionized water at a concentration of 0.1 M. Different concentrations of these inorganic salts were added to the colloidal Ag NPs solution, which was then centrifuged at 3500 rpm for three cycles, each lasting 9 min. The obtained sample was stored at 4 °C for further use.

#### 2.3.2. Fabrication of MXene (Ti_3_C_2_T_x_) @Ag NPs 

The SERS substrate was prepared using electrostatic self-assembly. First, the MXene (Ti_3_C_2_T_x_) surface was functionalized with a positively charged cationic polymer, PDDA. Specifically, 0.1 mg/mL MXene (Ti_3_C_2_T_x_) was mixed with PDDA at various concentrations (1 mg/mL, 0.5 mg/mL, 0.1 mg/mL, 0.01 mg/mL, and 0.005 mg/mL) and stirred for 3 h. The mixture was centrifuged at 3500 rpm for 60 min, followed by repeated washing and dispersion of the resulting precipitate in deionized water. Then, the citrate-synthesized colloidal Ag NPs solution was deposited onto the MXene/PDDA surface through electrostatic adsorption. PDDA-modified MXene (Ti_3_C_2_T_x_) was gradually added to the Ag NPs solution, with varying ratios between MXene (Ti_3_C_2_T_x_) and Ag NPs ranging from 1:130 to 1:250 while stirring continuously. After centrifugation at 3500 rpm for 15 min and several washing steps, the final MXene (Ti_3_C_2_T_x_) @Ag NPs was dispersed in deionized water for further use.

#### 2.3.3. Analyte Preparation and SERS Detection

The probe analyte, R6G, and the target analyte, furfural, were initially dissolved in deionized water and ethanol, respectively, to create stock solutions of specific concentrations. A series of standard R6G solutions with different concentrations were prepared (10^−4^ to 10^−9^ M), while furfural was diluted to concentrations ranging from 1 × 10^−2^ M to 5.2 × 10^−6^ M. The control MXene (Ti_3_C_2_T_x_) @Ag NPs SERS substrate was applied to a wafer slide for detection using a Raman microscope. SERS spectra were collected using a confocal laser Raman spectrometer with an excitation wavelength of 785 nm, a laser power of 25 mW, an integration time of 5 seconds per spectrum, and three scans. Additionally, the acquisition range was 600~2000 cm^−1^. Measurements were taken from at least five different positions to ensure accuracy.

#### 2.3.4. Statistical Analysis

All acquired SERS spectra were analyzed using Origin 2018 software, with each spectrum representing the average of five measurements taken from different positions. The results were then subjected to a one-way analysis of variance (ANOVA), with differences considered statistically significant at *p* < 0.05, as indicated by different lowercase letters (a–d). The ANOVA was performed using SPSS 26.0 software. 

## 3. Results and Discussion

### 3.1. Characterization of Materials

**Figure 1:**
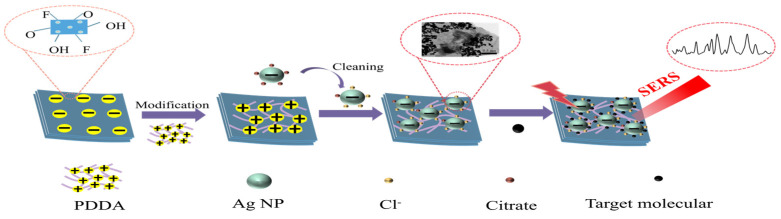
Schematic illustration of the fabricated process and detection of MXene/PDDA @Ag NPs.

**Figure 2:**
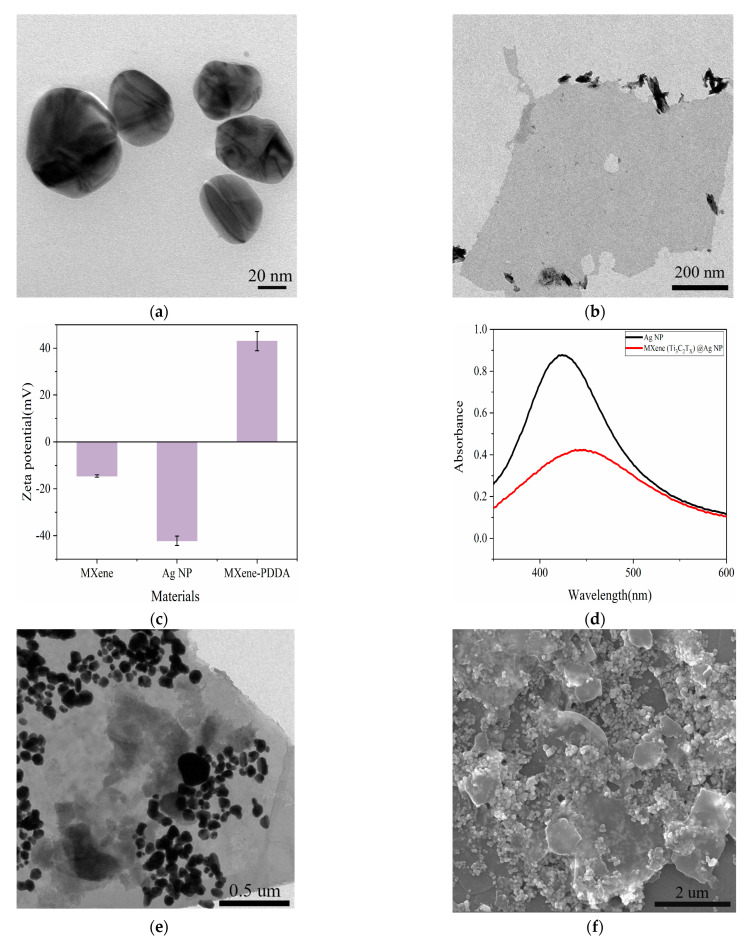
TEM images of (**a**) Ag NPs; (**b**) MXene (Ti_3_C_2_T_x_) nanosheets. Zeta potential of (**c**) pristine MXene (Ti_3_C_2_T_X_), Ag NPs, and PDDA-modified MXene (Ti_3_C_2_T_X_). UV-visible spectra of (**d**) Ag NPs and MXene (Ti_3_C_2_T_X_) @Ag NPs. TEM image of (**e**) MXene (Ti_3_C_2_T_X_) @Ag NPs. SEM image of (**f**) MXene (Ti_3_C_2_T_X_) @Ag NPs at the outer edge of the “coffee ring” structure.

This study proposed a strategy for fabricating MXene (Ti_3_C_2_T_X_) @Ag NPs composite materials as a SERS substrate. The fabrication process of the SERS substrate and the detection mechanism of the MXene (Ti_3_C_2_T_X_) @Ag NPs substrate are presented in Figure 1. This process resulted in a well-constructed MXene (Ti_3_C_2_T_X_) @Ag NPs SERS substrate, with uniformly distributed Ag NPs, forming high-density SERS “hot spots”. The SERS substrate was integrated with a Raman spectrometer for the rapid furfural detection in Baijiu. The TEM images in Figure 2a,b revealed Ag NPs with an average diameter of 50 nm and MXene (Ti_3_C_2_T_x_) nanosheets with lateral dimensions of approximately 400 nm, characterized by their ultrathin, single-layer, or few-layer structure. These findings demonstrated that the MXene (Ti_3_C_2_T_x_) nanosheets have a favorable surface morphology and reduced layer thickness. Previous studies have reported that double-sided adsorption is the primary mechanism for Ag NPs adsorption on the Ti_3_C_2_T_x_ surface [36]. The structural features of MXene (Ti_3_C_2_T_X_) nanosheets enabled the efficient loading of Ag NPs and enhanced electron interactions between them, leading to the formation of MXene (Ti_3_C_2_T_X_) @Ag NPs composite structures with densely packed “hot spots”.

During the construction of the composite structure, direct adsorption of Ag NPs onto the MXene (Ti_3_C_2_T_X_) surface was difficult due to the negative charge of MXene (Ti_3_C_2_T_X_) caused by functional groups like hydroxyl (-OH) and fluorine (-F) [37]. Similarly, citrate-coated Ag NPs also carried a negative charge. To resolve this issue, PDDA was used to modify the surface electrical properties of MXene (Ti_3_C_2_T_X_), resulting in a positively charged MXene (Ti_3_C_2_T_X_) surface [38]. The successful modification was confirmed through zeta potentialmeasurements. As shown in Figure 2c, MXene (Ti_3_C_2_T_X_) and Ag NPs exhibited negative zeta potentials, consistent with the previous observations. The introduction of PDDA led to a positive charge transformation in the MXene (Ti_3_C_2_T_X_) @Ag NP substrate, indicating the successful preparation of the modified substrate.

Additionally, the UV-visible absorption spectra of Ag NPs and MXene (Ti_3_C_2_T_X_) @Ag NPs were analyzed to identify the observed structural changes. For the 50 nm Ag NPs, a localized surface plasmon resonance produced a single absorption peak at 420 nm [39]. When Ag NPs were loaded onto PDDA-modified MXene (Ti_3_C_2_T_X_), the plasmon peaks broadened and redshifted towards longer wavelengths, as shown in Figure 2d. This shift was attributed to strong plasmon coupling between Ag NPs and modified MXene (Ti_3_C_2_T_X_), indicating the successful loading of Ag NPs onto the MXene (Ti_3_C_2_T_X_) surface. 

Consequently, MXene (Ti_3_C_2_T_X_) @Ag NPs were successfully synthesized with uniformly adsorbed Ag NPs on the few-layer surface of the MXene (Ti_3_C_2_T_X_) through electrostatic self-assembly. The interparticle gaps exhibited a relatively uniform distribution, indicating the successful fabrication of the SERS substrate, as shown in Figure 2e. Furthermore, a suspension of MXene (Ti_3_C_2_T_X_) @Ag NPs was dropped onto an aluminum foil-wrapped wafer slide, forming a distinct “coffee ring” pattern. The SEM image of the outer edge of the “coffee ring” pattern, shown in Figure 2f, revealed a uniform distribution of firmly anchored Ag NPs within the MXene (Ti_3_C_2_T_X_) @Ag NPs structure with dense “hot spots”.

### 3.2. Optimization of the Experimental Conditions 

#### 3.2.1. Optimization of the Aggregating Agent

**Figure 3:**
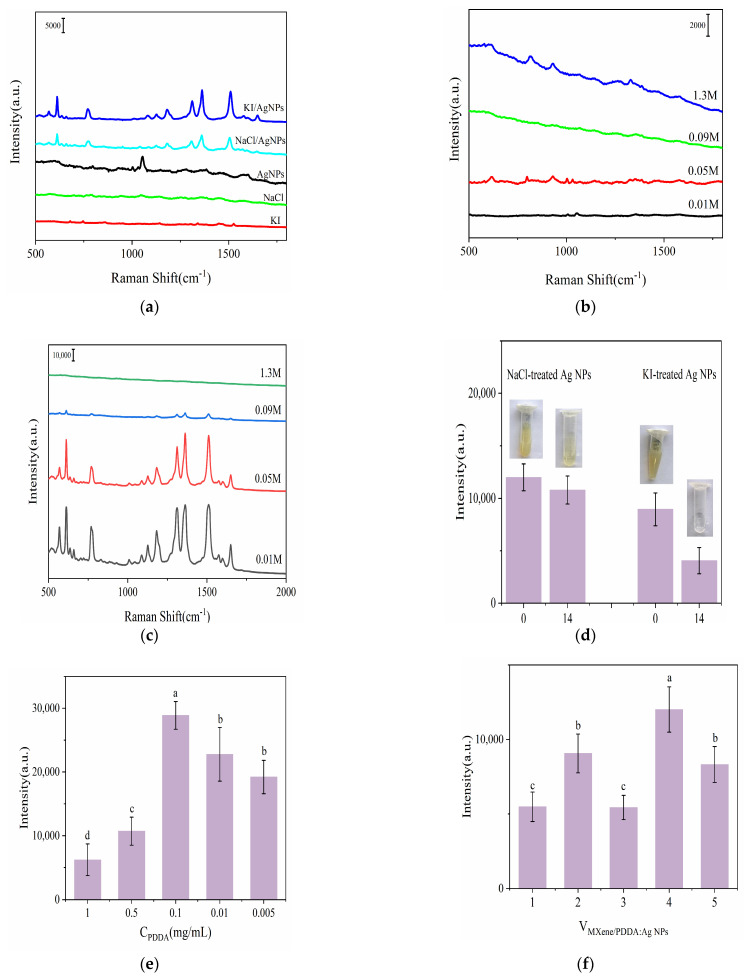
Raman spectra of (**a**) Ag NPs, (0.1 M) KI-treated Ag NPs, (0.1 M) NaCl-treated Ag NPs, and 1 × 10^−6^ M R6G dropped on NaCl-treated Ag NPs and KI-treated Ag NP SERS substrate; (**b**) different concentrations of NaCl-treated Ag NP SERS substrate (0.01 M, 0.05 M, 0.09 M, 1.3 M); (**c**) 1 × 10^−5^ M R6G dropped on NaCl-treated Ag NP SERS substrate with different concentrations of NaCl (0.01 M, 0.05 M, 0.09 M, 1.3 M); 1 × 10^−6^ M R6G Raman intensity of (**d**) the storge time of NaCl-treated Ag NPs and KI-treated Ag NP SERS substrates; (**e**) different concentrations of PDDA; 1 × 10^−7^ M R6G signal intensities of (**f**) the volume of ratio between MXene (Ti_3_C_2_T_X_) and Ag NPs. Different letters above the bars indicate significant differences (*p* < 0.05).

The selection of NaCl and KI for treating Ag NPs was based on their effectiveness in purifying the substrates and improving SERS performance. As shown in Figure 3a, NaCl and KI treatment resulted in purer substrates, with NaCl-treated Ag NPs exhibiting higher SERS signal intensity than those treated with KI. This difference is likely due to iodine ions having a significantly higher affinity for the surface of Au/Ag than anions such as chloride ions [40]. Additionally, cations formed “hot spots”, balancing the effects of competitive adsorption and steric hindrance. As a result, Na^+^ emerged as a better choice [41], while anions and target molecules coadsorbed, influencing SERS detection [42]. However, due to the competition between R6G molecules and specific anions, such as iodine and chloride for adsorption sites [43], NaCl was selected for subsequent experiments.

Subsequently, the concentration of NaCl was adjusted to 0.01 M, 0.05 M, 0.09 M, and 1.3 M for the treatment of Ag NPs, with R6G used as the probe molecule for Raman detection. As shown in Figure 3b, different NaCl concentrations led to a decrease or complete disappearance of the Raman peak corresponding to citrate ions in Ag NPs. The Ag NPs treated with 0.01 M and 0.09 M NaCl exhibited higher cleanliness compared to those treated with 0.05 M and 1.3 M NaCl, wherein only minimal characteristic peaks of citrate were observed (Figure 3b). Additionally, as indicated by the enhancement ability of Ag NPs with R6G, those treated with 0.01 M NaCl demonstrated superior SERS performance (Figure 3c). Furthermore, Figure 3d illustrates that the NaCl-treated Ag NPs maintained long-term stability than those treated with KI. This observation was confirmed by measuring the Raman peak intensities of both samples after 14 days of storage following treatment with NaCl or KI. The KI-treated samples changed color from grayish-green to transparent and experienced a significant decrease in Raman spectrum intensity. In comparison, the Ag NPs treated with 0.01 M NaCl maintained their color and Raman intensity, showing no noticeable changes over the same period. Therefore, 0.01 M NaCl was chosen for use in subsequent experiments. 

#### 3.2.2. Optimization of PDDA Concentration

The uniformity of the MXene (Ti_3_C_2_T_X_) @Ag NPs composite structure is crucial for enhancing detection sensitivity and repeatability. To achieve uniform distribution and strong bonding between MXene (Ti_3_C_2_T_X_) and Ag NPs, the concentration of PDDA, which acts as a bonding agent, was optimized. As shown in Figure 3e, as the concentration of PDDA decreased from 1 mg/mL to 0.1 mg/mL, the Raman signal intensity increased, reaching its peak at 0.1 mg/mL. However, beyond this concentration, the Raman signal decreased. The observed differences in results were statistically significant (*p* < 0.05). These phenomena could be attributed to the characteristics of PDDA, a high-molecular-weight cationic polymer with an elongated chain structure, which can introduce steric hindrance [44]. At PDDA concentrations below 0.1 mg/mL, there was insufficient PDDA for effective surface charge modification of MXene (Ti_3_C_2_T_X_), leading to reduced Ag NPs adsorption. Conversely, higher PDDA concentrations caused the excessive aggregation of Ag NPs within the MXene (Ti_3_C_2_T_X_) @Ag NPs composite. This aggregation disrupted the electrostatic repulsion between nanoparticles, causing fusion and subsequent disappearance of “hot spots”, which decreased the Raman signal intensity. Based on these findings, a PDDA concentration of 0.1 mg/mL was selected for subsequent experiments.

#### 3.2.3. Optimization of the Volume Ratio between MXene (Ti_3_C_2_T_X_) @Ag NPs and Ag NPs

The volume ratio between MXene (Ti_3_C_2_T_X_) and Ag NPs was further optimized in the subsequent experiment. As shown in Figure 3f, increasing the volume of Ag NPs resulted in a higher loading of Ag NPs onto MXene (Ti_3_C_2_T_X_) nanosheets, resulting in a higher density of “hot spots” and thereby enhancing the SERS signal. The optimal SERS signal was achieved with a 1:200 ratio of Ag NPs to MXene (Ti_3_C_2_T_X_). This efficiency was attributed to the complete and uniform coverage of Ag NPs on the MXene (Ti_3_C_2_T_X_) surface, which amplified the SERS signal through localized surface plasmon resonance between adjacent Ag NPs and charge transfer among the MXene (Ti_3_C_2_T_X_), target molecules, and Ag NPs. However, exceeding the optimal ratio caused a decrease in SERS signals, likely due to the uncontrolled agglomeration of excessive nanoparticles, which adversely affected the Raman signals. Therefore, a final optimized ratio of 1:200 was selected for subsequent experiments.

### 3.3. SERS Performance of MXene (Ti_3_C_2_T_X_) @Ag NPs

#### 3.3.1. Sensitivity of MXene (Ti_3_C_2_T_X_) @Ag NP Substrates

**Figure 4:**
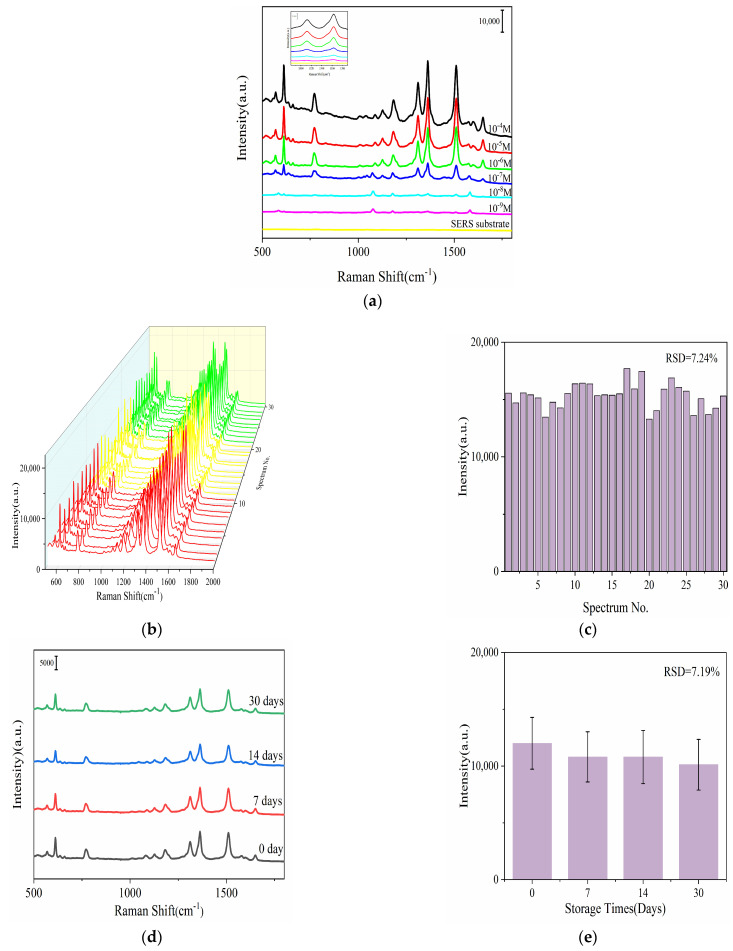

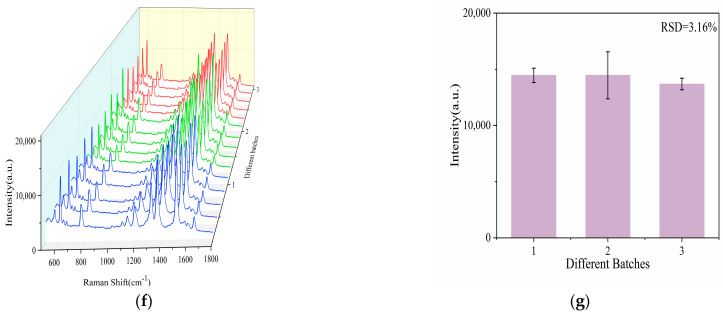
Raman spectra of R6G (**a**) at the SERS substrate. Raman spectra of 1 × 10^−6^ M R6G from (**b**) three SERS substrates. Raman intensities of 1 × 10^−6^ M R6G, on average, at 1364 cm^−1^ from (**c**) three SERS substrates. Raman spectra of 1 × 10^−6^ M R6G of (**d**) SERS substrates from different days. Raman intensities of 1 × 10^−6^ M R6G, on average, at 1364 cm^−1^ of (**e**) SERS substrates from different days. Raman spectra of 1 × 10^−6^ M R6G of (**f**) SERS substrates from different batches, and the same color is the same batch. Raman intensities of 1 × 10^−6^ M R6G, on average, at 1364 cm^−1^ of (**g**) SERS substrates from different batches.

The SERS performance of the MXene (Ti_3_C_2_T_X_) @Ag NPs substrates was evaluated under optimized conditions, demonstrating exceptional SERS enhancement capabilities. As shown in Figure 4a, the characteristic Raman spectra of R6G (612 cm^−1^, 774 cm^−1^, 1181 cm^−1^, 1364 cm^−1^, 1509 cm^−1^, 1574 cm^−1^, and 1649 cm^−1^) [45] were detected across concentrations from 10^−4^ M to 10^−9^ M, with a detection limit of 10^−9^ M. This sensitivity represented a two-orders-of-magnitude improvement compared to using Ag NPs alone as the SERS substrate. Moreover, the Raman enhancement factor (*EF*) was calculated to be approximately 7.08 × 10^5^ according to Equation (1) [46]. The obvious enhancement of the Raman signal when using MXene (Ti_3_C_2_T_X_) @Ag NPs as the SERS substrate can be due to the flat surface and abundant electronic properties of the MXene (Ti_3_C_2_T_X_) surface, which facilitated the complete absorption of Ag NPs and resulted in an increased density of plasmonic “hot spots”. This excellent property of MXene (Ti_3_C_2_T_X_) @Ag NPs is mainly attributed to plasmon coupling electromagnetic enhancement effects among nearby Ag NPs and charge transfer among MXene (Ti_3_C_2_T_X_), Ag NPs, and target molecules. Furthermore, the uniform surface of MXene (Ti_3_C_2_T_X_) provided abundant adsorption sites for analyte molecules, thereby improving the accuracy of detection.

(1) $$EF=\frac{I_{SERS}\times C_{\mathrm{Normal}}}{C_{SERS}\times I_{\mathrm{Normal}}}$$

*I_SERS_* and *I_Normal_* represent the intensities of the surface-enhanced Raman and normal Raman modes, respectively. *C_SERS_* and *C_Normal_* represent the analyte concentrations in the SERS and the normal Raman measurements, respectively. The enhancement factor calculation was based on the vibrational mode at 1364 cm^−1^.

#### 3.3.2. Repeatability, Uniformity, and Stability of MXene (Ti_3_C_2_T_X_) @Ag NPs Substrate

The uniformity of the MXene (Ti_3_C_2_T_X_) @Ag NPs substrate was evaluated by gathering SERS spectra from 30 randomly selected regions across three different spots. The relative standard deviation (RSD) at 1364 cm^−1^ was calculated. As shown in Figure 4b,c, the characteristic peaks of the R6G Raman spectra at 1364 cm^−1^ exhibited remarkable similarity, with an associated RSD of 7.24%, indicating favorable uniformity of the MXene (Ti_3_C_2_T_X_) @Ag NPs substrates. The stability of the MXene (Ti_3_C_2_T_X_) @ Ag NPs substrate was also investigated over time, with evaluations at 0, 7, 14, and 30 days. As shown in Figure 4d,e, the Raman intensities at 1311 cm^−1^ and 1364 cm^−1^ showed no significant after 30 days of storage, with an RSD of only 7.19%. These results demonstrated the excellent long-term stability of the substrate. Additionally, to verify the reliability of the SERS substrates across different batches, the repeatability was assessed using three distinct batches. As illustrated in Figure 4f,g, the MXene (Ti_3_C_2_T_X_) @ Ag NPs substrates consistently displayed comparable SERS signals, with an RSD value as low as 3.16%. This consistency indicated that the proposed substrate possesses excellent repeatability, uniformity, and stability.

### 3.4. Detection of Furfural Sample

**Figure 5:**
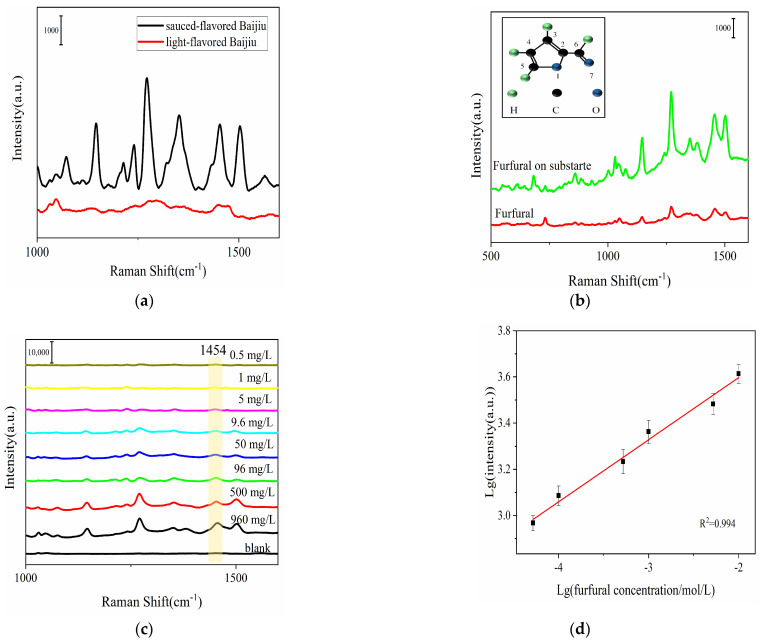
SERS spectra of (**a**) sauce-flavored Baijiu and light-flavored Baijiu (**b**) pure furfural and furfural on SERS substrate; (**c**) concentrations of furfural collected from MXene (Ti_3_C_2_T_X_) @Ag NP SERS substrate. Linear relationship between (**d**) logarithmic intensities of I_1453_ and logarithmic furfural concentrations.

The rapid detection of furfural in Baijiu poses a significant challenge. To address this, we proposed a strategy using the MXene (Ti_3_C_2_T_X_) @Ag NPs SERS substrate, employing the SERS method for furfural detection. This substrate amplified the Raman signal through both physical enhancements and charge transfer with furfural molecules, increasing their molecular polarizability and the Raman signal [47]. Furthermore, the negatively charged MXene (Ti_3_C_2_T_X_) modified with PDDA attracted the negatively charged furfural molecules via electrostatic attraction. These factors enable the highly sensitive detection of furfural in Baijiu. The Raman spectra of sauce-flavored Baijiu and light-flavored Baijiu are presented in Figure 5a. It can be observed that the sauce-flavored Baijiu exhibited a higher Raman signal upon deposition on the SERS substrate, indicating a high furfural content. In contrast, the light-flavored Baijiu showed minimal signal, serving as an appropriate blank sample for spike experiments. As shown in Figure 5b, the Raman intensity of furfural deposited on the SERS substrate exhibited a significantly higher Raman intensity compared to the directly obtained Raman signal of furfural without the SERS substrate. The results demonstrated that this SERS substrate effectively amplified the Raman signal. As shown in Figure 5c, the characteristic Raman peaks of furfural molecules were primarily observed at approximately 1366 cm^−1^, attributed to the in-plane bend of C6-H. Another peak around 1393 cm^−1^ corresponded to the in-plane ring breathing of furan ring with antisymmetric scissoring of C4-H, C5-H, C6-H, and C2-H. Additionally, a peak at 1474 cm^−1^ corresponded to the in-plane ring breathing of the furan ring with antisymmetric scissoring of C2-C3, C4-C5, C5-H, and C6-H. Finally, a peak at 1571 cm^−1^ signified the stretching motion of the carbonyl group (C=O) and the asymmetrical stretching of two carbon–carbon double bonds (C=C) in the furan ring of the furfural molecule [47,48,49]. The SERS intensities and furfural concentrations (ranging from 960.8 mg/L to 5 mg/L) exhibited a linear correlation, as shown in Figure 5d. The standard curve equation was y = 0.2688x + 4.1348, where x represents the logarithmic furfural concentration and y represents the logarithmic Raman intensity. The linear correlation coefficient was R^2^ = 0.994, with a limit of detection (LOD, S/N = 3) of 0.5 mg/L. Notably, the detection sensitivity achieved using the MXene (Ti_3_C_2_T_X_) @Ag NP substrate for furfural was 0.5 mg/L, surpassing previous reports in the literature. Additionally, the practicability of the proposed SERS substrate was demonstrated. As shown in Table 1, light-flavored Baijiu with different furfural concentrations (960 mg/L, 500 mg/L, 96 mg/L) was used for real sample detection, showing the recovery rates from 97.5% to 98.6% with RSD values ranging from 2.01% to 7.83%. This method demonstrated the reliable application of the MXene (Ti_3_C_2_T_X_) @Ag NPs substrate for detecting furfural.

## 4. Conclusions

In this study, a novel composite SERS substrate, MXene (Ti_3_C_2_T_X_) @Ag NPs, was developed to improve the rapid detection of furfural in Baijiu. The substrate demonstrated excellent SERS performance, which was attributed to the spatial enhancement mechanism from plasmonic coupling between nearby Ag NPs and charge transfer among the MXene (Ti_3_C_2_T_X_), target molecules, and Ag NPs. This influence enabled a detection limit as low as 10^−9^ M for R6G and an enhancement factor of up to 7.08 × 10^5^. Furthermore, the substrate demonstrated long-term stability, good uniformity (RSD = 7.24%), and repeatability (RSD = 3.16%) for the R6G molecule under optimized conditions. In addition, the SERS intensity also showed a strong linear correlation with furfural concentration, with a linear correlation coefficient of R^2^ = 0.994. It also achieved a limit of detection (LOD, S/N = 3) of 0.5 mg/L for furfural in Baijiu samples. Overall, this study significantly enhanced the sensitivity, reliability, and speed of furfural detection in Baijiu. It holds promising potential for detecting hazardous substances and distinguishing between authentic and counterfeit Baijiu products. Future research could further explore the potential of MXene (Ti_3_C_2_T_X_) as a versatile SERS substrate and incorporate stoichiometric analysis to achieve more robust results and enhance the limit of detection.

## Figures and Tables

**Table 1:** Recoveries of furfural in light-flavored Baijiu.

Simulated Sample	Added (mg/L)	Detected (mg/L)	Recovery (%)	RSD (%)
Light-flavored Baijiu	960	941.584	98.01%	5.78
	500	492.771	98.63%	2.01
	96	93.668	97.49%	7.83

n = 5.

## Data Availability

The original contributions presented in the study are included in the article, further inquiries can be directed to the corresponding author.
